# Late port-site metastasis of unexpected gallbladder carcinoma after laparoscopic cholecystectomy: A case report

**DOI:** 10.1097/MD.0000000000037880

**Published:** 2024-05-03

**Authors:** Abdullah Aloraini,, Khaled Alshehri, Rahaf Alshammari,, Abdulhakim Bin Onayq,, Mohammed Ayesh,, Malak Alzahrani,, Sulaiman A. AlShammari,, Faisal Alsaif,

**Affiliations:** a General, HPB & Transplant Surgeon, Department of Surgery, College of Medicine, King Saud University, Riyadh, Saudi Arabia; b Bachelor of Medicine and Bachelor of Surgery, College of Medicine, King Saud University, Riyadh, Saudi Arabia; c Department of Radiology, King Khalid University Hospital, King Saud University Medical City, Riyadh, Saudi Arabia; d Department of Pathology, College of Medicine, King Saud University, Riyadh, Saudi Arabia.

**Keywords:** histopathological, laparoscopic cholecystectomy, port-site metastasis, treatment of recurrence, unexpected gallbladder carcinoma

## Abstract

**Introduction:**

Incidental gallbladder carcinoma refers to a discovery of gallbladder cancer during or after cholecystectomy. Late port-site metastasis (PSM) following Laparoscopic cholecystectomy (LC) is rare with an incidence rate of 10.3%.

**Patient concerns:**

We report a case of a 58-year-old man who presented with a painful abdominal wall mass for 6 weeks. He had a history of LC for symptomatic cholelithiasis, 8 years prior.

**Diagnosis:**

Histopathological examination revealed a positive result for metastatic adenocarcinoma from the abdominal wall mass. Moreover, Positron emission tomography (PET) showed a small focus of intense fluorodeoxyglucose (FDG) uptake in the gallbladder bed, which was highly suspicious for malignancy.

**Intervention:**

Decision was to proceed with surgery owing to uptake in the gallbladder bed with single-site metastasis to the previous port site. In addition, in the board meeting, an agreement was reached for performing distal pancreatectomy with splenectomy owing to uncertainty of malignancy based on what was discovered during the full metastatic workup. Diagnostic laparoscopy followed by midline laparotomy performed. Radical completion cholecystectomy with lymphadenectomy was done. Followed by complete resection of the anterior abdominal wall. Distal pancreatectomy and splenectomy were then performed.

**Outcome:**

Pathological diagnosis showed metastatic/invasive, moderately differentiated adenocarcinoma with positive margins on the posterior surface of excised port-site mass. The positive margins necessitated further chemoradiotherapy, followed by adjuvant chemotherapy until lung metastasis was identified. After this, the patient was scheduled for palliative chemotherapy.

**Conclusion:**

Presence of PSM is often associated with peritoneal metastasis. For this reason, it is advised to evaluate the patient for possible metastasis.

## 1. Introduction

Laparoscopic cholecystectomy (LC) with routine histological analysis is the gold standard for the surgical treatment of benign and early-stage malignant gallbladder lesions. Gallbladder cancer (GBC) is a rare, malignant neoplasm of the biliary system.^[1]^ The majority of cases are diagnosed incidentally during or after cholecystectomy, representing 0.2% to 3% of histopathological findings post-LC.^[2]^ Cholelithiasis is the main risk factor for GBC. Though the process is not clearly understood, chronic irritation and inflammation of the gallbladder are thought to play a major role. Other factors associated with a higher risk of GBC include female sex, obesity, ethnicity, gallstone size, and geographical location.^[1,2]^ The incidence of GBC is particularly high in Chile and Southern India, where there are high rates of cholecystitis. Other high-rate regions include Poland, Pakistan, and Japan.^[1]^

The last decade has seen several reports of late port-site metastasis (PSM) of GBC. However, late PSM) following incidental GBC post-LC is rare with an incidence rate of 10.3%.^[3]^ The management and outcomes of late PSM are case dependent. We report a case of a 58-year-old man who presented with delayed PSM, 8 years following LC for biliary colic.

## 2. Case presentation

A 58-year-old man presented to the outpatient clinic complaining of a painful abdominal wall mass for 6 weeks. The patient reported the size of the mass to have been increasing for 8 months along with weight loss and loss of appetite. The patient had a history of LC for symptomatic cholelithiasis, 8 years prior, which was complicated postoperatively by biliary pancreatitis. Consequently, a pancreatic pseudocyst was discovered and managed successfully with endoscopic cystogastrostomy. Until this presentation, the patient had been in good health. The patient reported a family history of pancreatic cancer; his brother was diagnosed at the age of 50 years, and 2 of his uncles from his paternal side died from pancreatic cancer.

Upon abdominal examination, a hard tender mass was palpable at the right costal angle near the port site scar of the LC. The mass was ill-defined, not fixed to the skin, and measured 3 cm × 4 cm.

**Figure 1.**
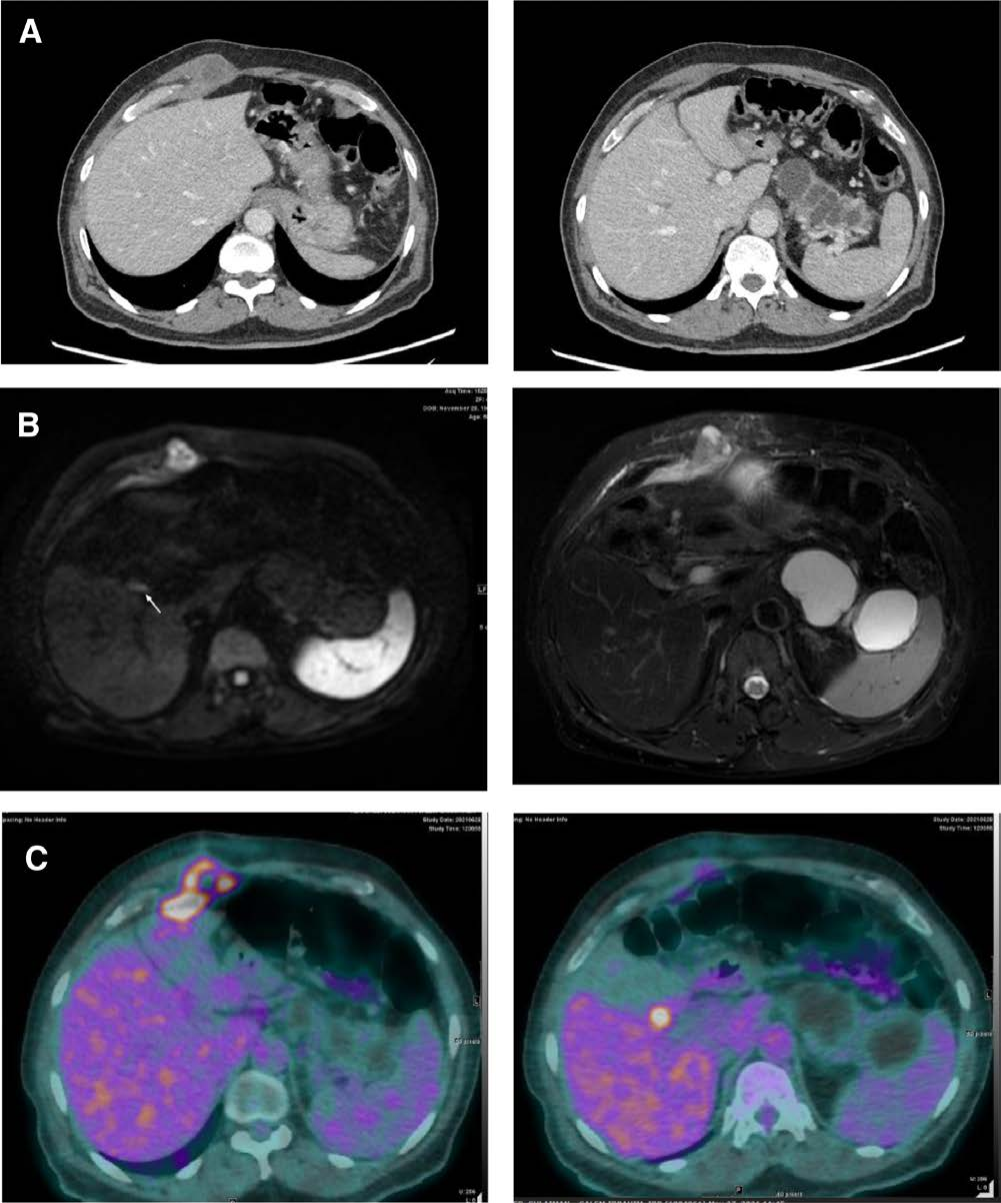
(A) Axial computed tomography CT scan with IV- contrast shows an anterior abdominal wall low density well-defined soft tissue mass at the right costo-chondoral area. The pancreas shows a significantly dilated duct as a sequelae of wall-off necrosis at the pancreatic neck secondary to pancreatitis. (B) Magnetic resonance imaging RI study of the liver in T2W and DW images show a focal soft tissue lesion of high signal intensity in T2W with diffusion restriction. Another tiny small linear high T2W signal and diffusion restriction are seen in the gallbladder bed. (C) Positron emission tomography computed tomography PET CT scan shows heterogeneous fluorodeoxyglucose (FDG) uptake in the right anterior abdominal wall small focus of intense FDG uptake in the gallbladder bed. CT = computed tomography, FDG = fluorodeoxyglucose, PET = positron emission tomography.

Laboratory tests were performed, and all results were within normal limits, except for an elevated carbohydrate antigen 19-9 (CA19-9) level of 15,339 units/mL, and a lipase level of 780 units/L. Computed tomography (CT) of the abdomen was used to characterize the abdominal wall mass and rule out any intra-abdominal pathology. CT showed that the pancreatic body and tail were replaced by a transversely oriented multiloculated cystic lesion taking the shape of the pancreas. The lesion measured 9.6 cm × 4.6 cm and was slightly infiltrating the splenic hilum. Another indeterminate small intramuscular soft tissue mass was identified in the right upper abdominal wall, measuring approximately 2.5 × 3 cm (Fig. 1A).

The patient was subsequently admitted for further investigation. Magnetic resonance imaging and magnetic resonance cholangiopancreatography revealed a stable cystic structure at the tail of the pancreas and splenic hilum and dilatation of the body and tail of the pancreatic duct. Moreover, the tail of the pancreatic duct had thin parenchyma. The soft tissue lesion in the right upper rectus abdominis muscle previously identified by CT was surrounded by inflammatory changes (Fig. 1B).

The case was discussed by a multidisciplinary team, and the decision was made to complete a full metastasis workup as there was no definitive primary origin. Colonoscopy and esophagogastroduodenoscopy were both normal. Positron emission tomography (PET) showed heterogeneous intense fluorodeoxyglucose (FDG) uptake in the right anterior abdominal wall mass; the biopsy proved it to be adenocarcinoma. PET also showed a small focus of intense FDG uptake in the gallbladder bed, which was highly suspicious for malignancy and compatible with the previously identified lesions on abdominal CT (Fig. 1C). CT of the chest was done as part of the metastasis workup and compared with a previous image from 9 years prior. The results showed multiple new subcentimeter pulmonary nodules. There was no uptake observed in the PET scan for the pulmonary nodules. As such, the decision was to proceed with surgery owing to uptake in the gallbladder bed with single-site metastasis to the previous port site. In addition, in the board meeting, an agreement was reached for performing distal pancreatectomy with splenectomy owing to an area of loss of enhancement at the neck of the pancreas with subsequent ductal dilatation, and uncertainty of malignancy.

**Figure 2.**
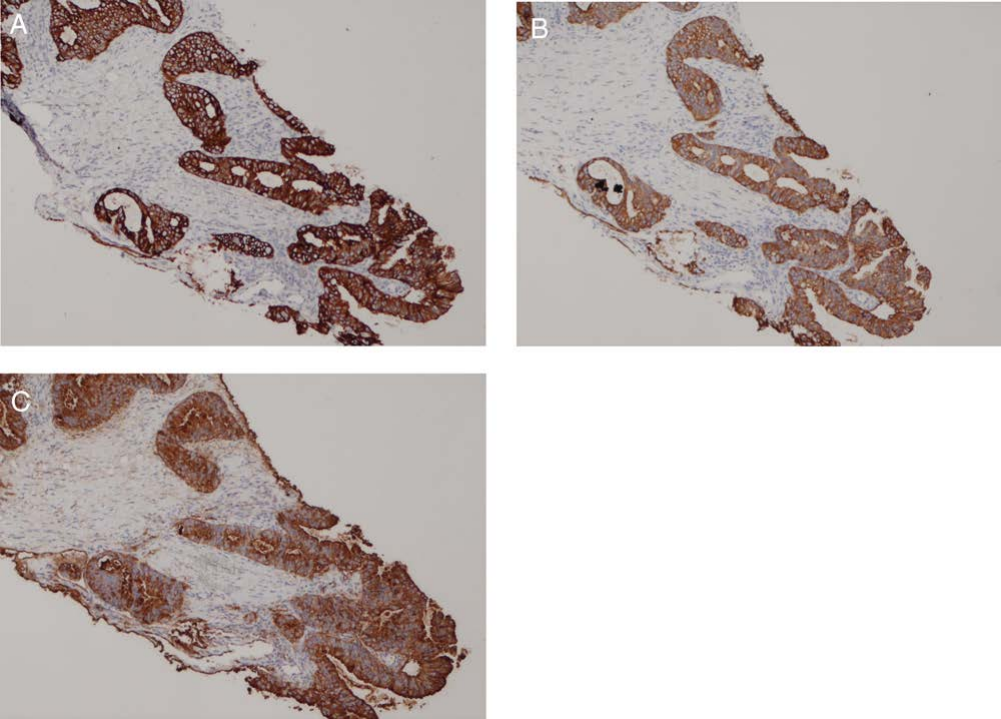
(A) Malignant cells are diffusely positive for CK7 (10X). (B) Malignant cells are positive for CK19 (10X). (C) Malignant cells are positive for CA19-9 (10X).

A tru-cut biopsy was taken from the abdominal wall mass and gave a positive result for metastatic adenocarcinoma. Tumor cells were positive for cytokeratin 7 (CK7), cytokeratin 19 (CK19), and CA19-9. (Fig. 2A, B, C) They showed patchy staining for caudal type homeobox 2 (CDX2) and focal staining for cytokeratin 20 (CK20), findings are all suggestive of a pancreatobiliary origin. A pathology report was requested for the gallbladder as this was performed in a peripheral hospital. The family provided the report, and a benign pathology was confirmed.

Diagnostic laparoscopy was first performed to rule out peritoneal metastasis; the results were negative. An upper midline laparotomy was then performed where the right upper quadrant and porta hepatis were explored, and remnants of the gallbladder and cystic duct were identified. Radical completion cholecystectomy with lymphadenectomy was performed. A frozen section of the proximal cystic duct margin was sent off for analysis and the result was negative for malignancy. Distal pancreatectomy and splenectomy were then performed, including the pseudocyst. The abdominal wall mass was assessed and was adherent to the costal margin. The mass and skin on top were excised completely and were shaved off the underlying rib. The abdominal wall defect was reconstructed using a vicryl mesh. The patient spent 3 days in the intensive care unit after surgery before returning to a regular ward. The postoperative course saw no complications. The patient was discharged in a stable condition, on day 8.

**Figure 3.**
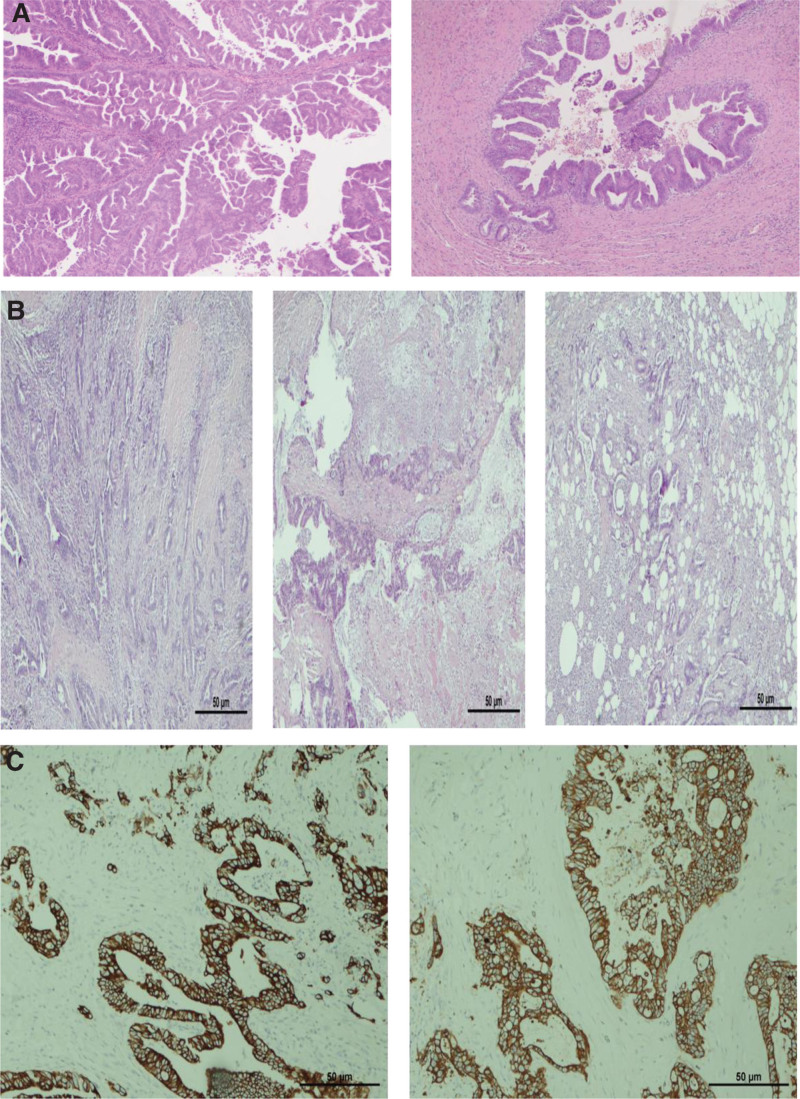
(A) Invasive biliary adenocarcinoma with a complex micropapillary pattern (H&E, 10X). (B) Excised soft tissue mass from abdominal wall which is infiltrated by a moderately differentiated adenocarcinoma of biliary origin (H&E, 10X). (C) Immunohistochemical stains show that the malignant cells are diffusely positive for CK7 (1) and CK19 (2) (IHC, 10X).

Several specimens from the cystic duct, porta hepatis lymph node, and abdominal wall mass were sent off for analysis. Firstly, the cystic duct showed both in situ and invasive biliary adenocarcinoma, with a microscopic focus of residual adenocarcinoma from the sutured resection margin of the cystic duct. However, this focus was not present in the frozen section side, suggesting that the tumor invaded parts of the muscular layer but had not reached the surrounding fat or adherent hepatic tissue. (Fig. 3A) The excised porta hepatis lymph node was reactive and negative for malignancy. The abdominal wall mass showed a fibrofatty and muscular tissue infiltrated by moderately differentiated adenocarcinoma of biliary origin with microscopic positive margin on the posterior surface. (Fig. 3B) Immunohistochemical staining was strong and diffuse for CK7 and CK19 and focal and mild for CK20 and CDX2. (Fig. 3C) This neoplasm was pathologically staged as T1 NO M1.

**Figure 4.**
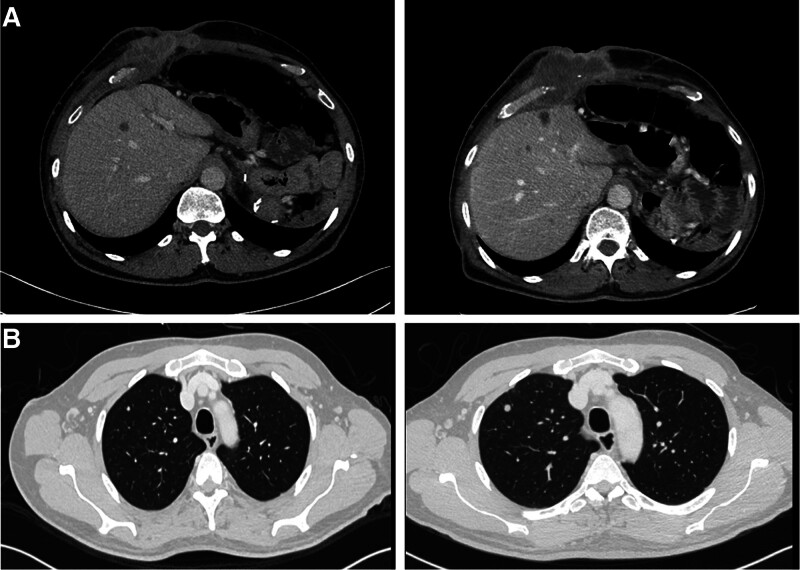
(A.1) Axial CT scan shows interval recurrence at surgical bed with new liver lesion. (A.2) Axial CT scan shows interval progression at surgical bed with new liver lesions. (B.1) Axial CT scan lung window setting shows a new lung nodule seen in the right upper lobe. (4B.2) Axial CT scan lung window setting shows interval increase in size of the right upper lung nodule. CT = computed tomography.

The case was again discussed by the multidisciplinary team. Owing to the positive margin of the excised port-site mass, chemoradiotherapy was recommended. The patient was subsequently scheduled for concurrent chemoradiotherapy (CCRT) for 5 weeks, followed by 4 months of high-dose capecitabine. After finishing CCRT and 2 cycles of capecitabine, an interval chest/abdomen CT was performed. The results showed an increase in the size and number of pulmonary nodules with an interval recurrence at surgical bed with a new liver lesion. (Fig. 4A1/4B) These findings raised the concern of potential intrathoracic metastasis; thus, a biopsy of the mass was warranted. The patient was scheduled for video-assisted thoracic surgery, though he developed deep vein thrombosis twice whilst awaiting surgery. The deep vein thrombosis was managed by anticoagulants and an inferior vena cava filter. Video-assisted thoracic surgery wedge resection of the pulmonary nodules was then performed. Multiple other nodules were identified intraoperatively. The postoperative pathology report showed similar morphology to that of biliary adenocarcinoma. The patient was started on Gemcitbine and Cisplatin but with irregularities and noncompliance with his chemotherapy appointments. Repeated imaging showed interval progression at surgical bed with new liver lesions (Fig. 4A2), and for that reason, the case was again discussed by the multidisciplinary team, and the decision was made to shift his treatment to another line of chemotherapy protocol: #4 FOLFOX. Repeated scans showed disease progression and new bilateral PE 2 months later. Then the patient was started on regorafenib which he was tolerating well with no further complaints. After 17 months post-surgery, the patient died having diffuse metastasis with local recurrence. The patient consented to the case report.

## 3. Discussion

GBC is relatively rare, despite it being the most common and aggressive type of biliary tree malignancy, accounting for approximately 60% of all cancers.^[4]^ The prevalence of incidental GBC has increased in recent years owing to the widespread increased use of LC. Reports show that 0.2% to 3% of the pathologies post-LC were incidental gallbladder cancer findings, accounting for 5.4% of all GBC cases.^[2,5]^ The decision to send all gallbladder specimens for histopathological analysis has been discussed among researchers. Subsequently, a new study recommended a selective approach, where histopathological analysis would be indicated only if an abnormal macroscopic finding was noticed or in the presence of risk factors.^[6]^ However, a further 2 studies in areas where the incidence of GBC is higher conclude the importance of examining all specimens regardless of their appearance.^[7,8]^ In our patient, the histopathological analysis for the gallbladder was performed in a different hospital and reported as a benign pathology.

Effective management of patients who present with incidental GBC is dependent on a detailed histopathological examination of the entire specimen. This enables both the depth of the invasion and surgical margins to be determined. For example, T1a with negative margins can be managed with simple cholecystectomy followed by observation, while an extended cholecystectomy, extensive hepatectomy, bile duct resection, and lymph node resection are the treatment choices for T2 and T3 invasive gallbladder cancer.^[9–11]^ The stage at the time of diagnosis ranges from 47% as stage T2 to 25.1% and 23% as T3 and T1, respectively.^[12]^

PSM following LC has been reported in many cases in the literature. However, late-type PSM is rare.^[3]^ Typically, patients present with PSM within 4 to 10 months after LC.^[2]^ The longest period till PSM occurrence was observed 12 years post-surgery.^[13]^ In our case, the patient presented 8 years after LC. However, the initial gallbladder histopathological analysis was not performed in our hospital and reported as a benign pathology. The prognosis of PSM is poor with the survival time averaging 10 months after recurrence.^[14]^ Razontovic et al, reported a 61-year-old female who presented with PSM post-surgery and died after 2 months.^[15]^ Conversely, Sandor et al, presented a case of PSM of GBC that was treated and followed up 11 months post-surgery with no further complaints.^[16]^

Ninety percent of PSM cases were found in the trocar site, from where the gallbladder specimen was extracted.^[3]^ The mechanisms underlying PSM are attributed to multiple factors including pneumoperitoneum, a perforated gallbladder wall, and contamination from the outer layer of the gallbladder.^[17]^ Preventive measures can be taken during surgery in patients with suspected GBC such as meticulous dissection of the gallbladder to avoid gallbladder perforation and bile spillover. Furthermore, the gallbladder should be extracted using an extraction bag.

The presence of PSM is often associated with peritoneal metastasis. For this reason, it is advised to evaluate the patient for possible metastasis. PET plays an important role in both diagnosis and management, guiding toward the primary tumor in cases where this is unknown. PET also enables the detection of further metastases. However, diagnostic laparoscopy is a crucial first step in preventing unnecessary intervention. Tumor staging and distant metastasis are the main deciding factors in the management plan.^[18]^ In our patient, PET showed localized lesions that were resectable, with tumor biology of slow growth. Based on this and the possibility of pancreatic cancer, the surgical treatment option was chosen. Although the presence of PSM considered as a stage IV gallbladder cancer and mandates systemic chemotherapy prior surgery, the case was discussed by the multidisciplinary team and the decision was made to proceed with upfront surgical resection based on the given favorable factors. Patient was started on adjuvant chemotherapy until lung metastasis and disease progression was identified. After this, the patient was started on regorafenib which he was tolerating well with no further complaints. Unfortunately, patient died after 17 months from surgery due to extensive local disease recurrence.

## Author contributions

**Data curation:** Khaled Alshehri, Malak Alzahrani.

**Formal analysis:** Malak Alzahrani.

**Investigation:** Khaled Alshehri.

**Methodology:** Khaled Alshehri, Rahaf Alshammari, Abdulhakim Bin Onayq, Sulaiman AlShammari.

**Project administration:** Abdullah Aloraini, Khaled Alshehri, Sulaiman AlShammari.

**Supervision:** Abdullah Aloraini, Khaled Alshehri, Sulaiman AlShammari, Faisal Alsaif.

**Visualization:** Mohammed Ayesh.

**Writing – original draft:** Khaled Alshehri, Rahaf Alshammari, Abdulhakim Bin Onayq, Sulaiman AlShammari.

**Writing – review & editing:** Khaled Alshehri, Rahaf Alshammari, Abdulhakim Bin Onayq, Sulaiman AlShammari.
